# The impact of COVID-19 on chronic pain

**DOI:** 10.3389/fpain.2023.1234099

**Published:** 2023-08-22

**Authors:** Abraham Lavin, Félix LeBlanc, Antonios El Helou

**Affiliations:** ^1^ Faculty of Medicine, Université de Sherbrooke, Sherbrooke, QC, Canada; ^2^ Division of Neurosurgery, Horizon Health Network, Moncton, NB, Canada; ^3^ Faculty of Medicine, Memorial University of Newfoundland, St. John's, NL, Canada

**Keywords:** COVID-19, chronic pain, pathophysiology and mechanism, therapeutic options, pandemic

## Abstract

A reduced quality of life is often a hefty burden that those with chronic pain are left to bear. This review of literature from PubMed, Google Scholar and other relevant studies focuses on the complex relationship between COVID-19 and chronic pain, which is challenging to study during the COVID-19 pandemic. In this review, we will briefly discuss the epidemiologic facts and risk factors, followed by the proposed pathophysiologic mechanisms. Furthermore, we will cover the therapeutic avenues regarding various molecules and their possible interactions, with the most promising being those whose mechanism of action can be directly linked to the pathophysiologic aspects of the condition. Finally, we will describe how to deal with a chronic pain patient who consults during the pandemic.

## 1. Introduction

The COVID-19 pandemic took the world by storm. This global crisis affected many dimensions of peoples’ lives, such as mental health and wellbeing due to isolation and loneliness, job loss and financial instability, and illness and grief (1). Vulnerable populations, such as people living with chronic pain, were impacted significantly. COVID-19 had complex effects on both current and newly created pain, much like the illness itself (1). Notably, there was an increase in “pain”-related search phrases globally, indicating a greater public interest and concern during the pandemic (2). Employment insecurity, social isolation, and recommendations concerning physical distancing had burdened people in numerous nations, all of which possibly contributed to their psychological distress and physiological pain (3, 4).

Identifying the risk factors associated with any health condition is important to prevent its development (5). Some domains have been identified as contributors to the potential development of chronic pain among hospitalized COVID-19 patients (5). First, the risk of post-traumatic stress disorder, social isolation both during and after discharge, and psychological burdens particular to the pandemic are all related to mental health burdens, which can be potential risk factors for chronic pain development in COVID-19 patients (5). Subsequently, the neurological manifestations caused by COVID-19 infection have been also identified as possible risk factors (5). Moreover, intensive care unit–associated risks, such as prolonged ventilation and immobility, repeated prone position, neuromuscular block, and sepsis or procedural pain, are all considered chronic pain risk factors (5). There are also COVID-19 patient–related factors embodied by a high comorbidity prevalence and an elderly population (5). In addition, acute pain associated with COVID-19 infection is also a risk factor for chronic pain development (5, 6). Finally, the challenges linked to rehabilitation services have been reported to contribute to the risk of chronic pain development. These challenges include potentially overworked rehabilitation services, poorly planned rehabilitation pathways, resource diversion from subsequent waves, insufficient concrete rehabilitative evidence related to COVID-19, fatigue, and multimorbidities (5).

In a controlled cross-sectional study, Soares et al. (7) compared 46 patients, who were discharged from the hospital following COVID-19, to a control group consisting of 73 patients, who were hospitalized during the same period but for reasons other than COVID-19. They demonstrated that *de novo* pain was significantly more prevalent in the COVID-19 group (65.2% vs. 11.0%, *p* = 0.001). In addition, 19.6% of the COVID-19 patients had new-onset chronic pain, compared to 1.4% (*p* = 0.002) of the control group. Thus, the study concluded that *de novo* chronic pain and new-onset pain were generally more prevalent in COVID-19 patients. Considering all of this, we realized the importance of studying chronic pain in patients with COVID-19, hence the relevance of this literature review.

## 2. Literature search

We searched for the keywords “COVID-19,” “chronic pain,” “pathophysiology and mechanism,” “therapeutic options,” and “pandemic” in PubMed and Google Scholar in May 2023 and then evaluated the present body of literature, considering all articles published before this date. The initial search yielded 1,131 articles, of which 56 were retained after the titles were analyzed,. We then analyzed these articles based on the abstract, type of study, and publication date to determine their relevance, of which 31 were retained. We included the English versions of articles including those written in foreign languages, mainly systematic reviews and some experimental studies that elucidated information pertinent to the keywords mentioned. In some instances, definitive websites and bibliographies of the selected articles were consulted for complementary information purposes.

## 3. Pain in COVID-19

Guerrero et al. (5) proposed a triage of patients into three diverse groups to demonstrate the disproportion in how COVID-19 affects different patients. The first group included those whose chronic pain first prevailed after COVID-19 infection, which may be categorized as a variety of post-viral chronic pain in which the pain is either directly related to organ damage caused by the acute infection or an entity referred to as long COVID-19 (8). Long COVID-19 is defined in the current literature as a post-viral syndrome, which comprises sleep disorders, chronic fatigue, and diffused myalgia (9, 10). This syndrome occurs in those who recover from acute COVID-19 infection with prolongation of the previously described symptoms for over 4 weeks (10). The pathology for this syndrome is not well defined and comprises primary symptoms related to the cardiovascular, neurological, psychological, and pulmonary systems such as, but not limited to, brain fog and other cognitive deficits, disordered sleep patterns, autonomic disturbances, dyspnea, anosmia, chest and joint pains, cardiac arrhythmias, and neuropathies (10). No diagnostic tools are currently available for this syndrome (10). The second group included those with exacerbating pre-existing chronic pain, most likely as a result of the pathophysiologic mechanisms described further (5). The third group included those who were feeling well before the pandemic but have since developed chronic pain. This group was mostly tied to the biopsychosocial model described below, as well as the various predisposing risk factors that were mentioned earlier (5).

## 4. Pathophysiology and mechanism

Shanthanna et al. (1) proposed a model that categorizes the reasoning into three main categories, i.e., systemic immune-inflammatory mechanisms, secondary mechanisms due to COVID-19 pathology or its associated treatments, and direct neuropathic mechanisms.

To begin, COVID-19 is thought to be neurotropic and capable of infiltrating host cells via the angiotensin-converting enzyme 2 (ACE2) receptors, which are present in the glial cells and neurons in the brain stem and regions, such as the paraventricular nucleus, rostral ventrolateral medulla, nucleus tractus solitarius, and subfornical organ (11, 12). Once it has infiltrated, there is a resulting microglial activation and astrogliosis which originate an extensive neuroinflammatory cascade, while the systemic inflammation associated with the infection disrupts the blood–brain barrier, causing subsequent extensive disruption of homeostasis within the brain and associated neuronal cell death (11). This systemic inflammation, which is characterized by a cytokine storm implicating interleukin-6, interleukin-10, and tumor necrosis factor-α, among others, results in the upsurge damage of various structures manifesting as joint pain and myalgia and other tissue pain observed in the new-onset chronic pain group (13, 14).

Studies have represented the implication of two pathways in pain, namely, the angiotensin-converting enzyme/angiotensin II/angiotensin II type 1 receptor (ACE/Ang II/AT1) as a pain transmission promoter in the dorsal horn and ACE2/angiotensin (I–VII)/Mas receptor as a pain moderator via the p38 mitogen-activated protein kinase phosphorylation inhibition (14–16). Studies have suggested that the ACE2 implication within the microglia and neurons of a mouse spinal dorsal horn led the group to hypothesize that the COVID-19 virus may infect ACE2-positive cells within the human spinal dorsal horn, culminating in a functional downturn of ACE2 that would then further cause an Ang II accumulation and a subsequent Ang (I–VII) depression and thereupon explain the possibility of SARS-CoV-2 spinal cord infection installation of pain (14, 16).

There are other proposed methods of entry, such as the one by Wu et al. (17) who described a blood circulatory pathway in which SARS-CoV-2 can directly infect the central nervous system, which ultimately culminates in the same result as the previously described. The implication of the nervous system explains the manifestations of fatigue symptoms, which are similar to those seen in chronic fatigue syndrome or myalgic encephalitis (18, 19). Impacts on chronic pain may further be explained by pathologic immune mechanisms, such as resident astrocyte and microglial stimulation along with leukocyte activation (20, 21).

In their rat model study, Moutal et al. proposed that the neuropilin-1 receptor (NRP-1) is the key to the pathophysiologic mechanism as its activation results in neurotropism (13, 22). They described how the viral spike protein binds to the NRP-1 receptor, blocking the NRP-1 binding and vascular endothelial growth factor-A (23). This model also unveils anti-allogenic properties, which could advocate for the role of increased transmission as explained by diminished pain symptoms (23).

Hyperinflammation and dysautonomia may be seen predominantly in patients with comorbidities due to the involvement of the autonomic nervous system, particularly the sympathetic nervous system (22, 24–26).

Secondary mechanisms due to COVID-19 pathology or its associated treatments encompass many syndromes as a direct result of the virus’ impact on chronic pain, such as that seen in intensive care unit admissions which may be analogous with post-intensive care-like syndrome and other secondary causes of pain (27). There is also evidence of an elevated stroke incidence among COVID-19 patients, as reported by Siepmann et al. (28) who conducted a meta-analysis of 165 patients, which may be a factor in the involvement of an increased post-stroke pain in those who survive. Finally, prolonged immobilization (>21 days) in prone ventilation positioning can result in other injuries such as peripheral nerve damage (29).

When it comes to analyzing the mechanism between COVID-19 and chronic pain, the current literature presents various options. A well-established model hypothesizes chronic pain as a biopsychosocial phenomenon that embodies a trident of biological, psychological, and social factors, all of which heavily influence and affect one another (30). With this model in mind, Guerrero et al. (5) proposed that given that the COVID-19 pandemic caused a prolonged state of not only financial but also social stress among many patients, this could not only cause a magnification in the prevalence of chronic pain but could also cause an aggravation of painful symptoms in those with pre-existing chronic pain. The group further goes on to establish that patients with known chronic pain were then placed in a difficult predicament, which consists of financial and personal loss, a new-found state of isolation, and new avenues needed to procure medical care which led to pain exacerbation (5).

## 5. Management and therapeutic options

Understanding the pathophysiology of acute post-COVID-19 pain has led to the proposal of various therapeutic options, such as the nervous system involvement and proinflammatory cytokine secretion, which point toward using low-dose naltrexone as a possible therapy for the perceived chronic pain as explained by its mechanism of influencing the release of the proinflammatory cytokines^1^ (1, 31, 32). Opioids have been reported to have menacing side effects such as endocrine changes and immune system suppression (33). While the clinical implication of this remains uncertain, the idea that patients receiving opioids for chronic pain may be at a higher risk for COVID-19 and other infections is supported by the observational studies explaining the increased infection prevalence within this group (34, 35). Non-steroidal anti-inflammatory drugs (NSAIDs) have been used for chronic pain modulation (36). Based on the proposed mechanism that NSAIDs inhibit the cyclo-oxygenase enzymes (COX-1 and COX-2), there was an assumption that NSAIDs could lead to an increased ACE and, thus, the intensity of the SARS-CoV-2 infection, prompting the French health minister to urge against the use of NSAIDs (34, 37). Although NSAIDs can obscure early infection symptoms such as myalgias and fever, such recommendations have since been refuted by various regulatory bodies^2^^,^^3^ (34, 38, 39). An expert panel has since recommended that all patients currently receiving NSAIDs continue their medication with prompt monitoring for side effects and report myalgias and mild fevers (34). Lower antibody levels were found in post-vaccination immunocompromised patients supporting the concern about the efficacy of patients receiving steroids and either the mRNA-based vaccine mRNA-1273 (Moderna) and BNT162b2 (Pfizer) or the viral vector AstraZeneca and Johnson & Johnson vaccine (40, 41). This concern is supported by the findings of Naranbhai et al. who discovered lower antibody levels in post-vaccination patients using steroids for cancer in a prospective cohort study that compared 1,001 infected patients to 1,638 control patients (42). These facts led The Faculty of Pain Medicine of the Royal College of Anaesthetists to advocate for caution regarding steroid injections throughout the COVID-19 pandemic^4^ (43).

Treating those with chronic post-COVID-19 pain and the mechanism of sympathetic nervous system overactivity has led to the deliberation of using stellate ganglion blocks to effectively treat the presenting chronic pain (24, 25). The sympathetic stellate ganglion, which is located in the lamina prevertebral fasciae cervicalis at the height of the first rib head, affects the function of the areas that it supplies, such as the brain, lung, neck, upper extremity, heart, and vessels comprising endothelial function, interstitium, immune system, and microcirculation (24). The stellate ganglion modulates both brain areas involved in the governing processes of the immune system and the immune processing in the periphery, explaining its influence in areas beyond its immediate supply such as the intestine (24). During an immunological response, the immune system and the autonomic nervous system interact quickly, resulting in an inflammation caused by the onset of an immune system cytokine storm and associated tissue damage by sympathetic hyperactivity (24). With this mechanism in mind, the stellate ganglion block's modulation of the sympathetic immune system would reduce signals to the areas and functions that it supplies (mentioned above), thus modulating pain (24).

Finally, treating the group of patients who had chronic pain and developed one or several flare-ups in relation to the COVID-19 pandemic, either by direct post-viral infection or by the psychological burden of the general pandemic, led to greater utilization of the biopsychosocial model (34). This model includes the ability to obtain care from social workers, physical therapists, and psychologists to adequately manage pain (34). During the pandemic, many of these services were adapted to sanitary circumstances via telemedicine as demonstrated by the National Health Service in the United Kingdom, which adopted the use of Microsoft Teams to facilitate communication among professionals and patients, or by the provincial Ministries of Health in Canada^5^, which loosened the regulations surrounding telemedicine (34, 44).

With regard to the impact of COVID-19 on chronic pain, it is not only important to have the therapeutic arsenal explained above at one's disposal but also, if not more important, to know when to use the proper treatment and have a solid plan when tackling this complex situation, and in this optic, a consensus within an international expert panel proposes a distinct model (34). In this suggested model, the first step to treat a patient with known chronic pain before the pandemic would be to arrange a phone call to establish the urgency of the consultation. If non-emergent, telemedicine visits should be scheduled when possible to avoid further aggravation, or virtual prescriptions should be issued if deemed necessary in the given context (34). If semi-emergent, case-by-case shared decision-making seems an acceptable approach (34). Finally, if urgent, an in-person consultation is justified, for example, in the case of an urgent procedure such as an intrathecal pump or neuromodulation malfunction or infection (34). Patients with pandemic-induced chronic pain could be treated by this model in the event of an acute exacerbation, but for long-term management, they should consult their regular healthcare provider and seek advice on which of the abovementioned therapies should be employed (34).

## 6. Discussion

The key to understanding the complex relationship between chronic pain and COVID-19 is the understanding of the underlying pathophysiologic mechanism, particularly the three main categories, i.e., systemic immune-inflammatory mechanisms, secondary mechanisms due to COVID-19 pathology or its associated treatments, and direct neuropathic mechanisms (1). The recent acknowledgments of the key receptors, such as NRP-1, the ACE/AngII/AT1, and ACE2/angiotensin (I–VII)/Mas receptor, along with the elucidation of the biopsychosocial model all facilitate the understanding of this phenomenon (14–16, 23, 30). Understanding these mechanisms allows elucidation of proper therapeutic options based on their mechanism of action to counter the pathophysiology, in particular, naloxone, NSAID, or the stellate ganglion block^6^ (24, 25, 31, 36).

The definition of the targeted population is equally important and can be described by three groups. The first group consists of those whose chronic pain first prevailed after COVID-19 infection caused by direct organ damage sustained during acute infection manifested by post-viral chronic pain, which is referred to as long COVID-19 (8). The second and third groups consist of those who had pre-existing chronic pain and those who were well before the infection and have since developed chronic pain, respectively, with the last group being closely tied to the predisposing risk factors and the biopsychosocial model previously outlined (5).

In May 2023, the World Health Organization (WHO) issued a press release in which the Director-General announced that COVID-19 is an ongoing and established health issue and is no longer considered a public health emergency of worldwide concern^7^ (45). Despite this announcement, there is yet to be a clear and precise strategy going forward on how to systematically approach the dilemma of the impact of COVID-19 on chronic pain.

## 7. Conclusion

In conclusion, COVID-19 has caused a profound impact by creating a new post-viral and persisting post-pandemic chronic pain syndrome and a large impact on those already living with chronic pain in many ways, often disproportionately. Some key characteristics are present within the patients that could predispose them to different outcomes when they are exposed to SARS-CoV-2. Understanding the implicated pathophysiologic mechanisms is key to not only understanding the clinical manifestation of the infection but also shedding light on some therapeutic options. Treatment will be often multidimensional to address all aspects of the condition and is essential to acknowledge the proper situation to use the appropriate therapy. Considering all the above, we recognize that conducting research with primary data such as an experimental study could be beneficial in providing further insights into this field. It is also noteworthy that findings cannot be generalized, but after the pandemic ended, it was declared that chronic pain will continue to be an issue; therefore, a multicenter prospective study is required to have accurate data and management plan.

## Footnotes

^1^
https://clinicaltrials.gov/ct2/show/NCT04756128.

^2^
https://www.fda.gov/drugs/drug-safety-and-availability/fda-advises-patients-use-non-steroidal-anti-inflammatory-drugs-nsaids-covid-19.

^3^
https://www.ema.europa.eu/en/news/ema-gives-advice-use-non-steroidal-anti-inflammatories-covid-19.

^4^
https://fpm.ac.uk/covid-19-fpm-response-concerns-over-safety-injected-steroids-pain-procedures.

^5^
https://www.healthcareitnews.com/news/emea/nhs-staff-receive-free-access-microsoft-teams-and-locum-s-nest.

^6^
https://clinicaltrials.gov/ct2/show/NCT04756128.

^7^
https://www.who.int/news/item/05-05-2023-statement-on-the-fifteenth-meeting-of-the-international-health-regulations-(2005)-emergency-committee-regarding-the-coronavirus-disease-(covid-19)-pandemic.
